# Assessing the Accessibility of Home-Based Healthcare Services for the Elderly: A Case from Shaanxi Province, China

**DOI:** 10.3390/ijerph17197168

**Published:** 2020-09-30

**Authors:** Xiaodong Di, Lijian Wang, Xiuliang Dai, Liu Yang

**Affiliations:** School of Public Policy and Administration, Xi’an Jiaotong University, No 28 Xianning West Road, Xi’an 710049, Shaanxi, China; dxd6794860@stu.xjtu.edu.cn (X.D.); xiuliangdai@stu.xjtu.edu.cn (X.D.); yangliu777@stu.xjtu.edu.cn (L.Y.)

**Keywords:** healthcare service for the elderly, accessibility, index system, principal component analysis

## Abstract

With the rapid increase of the elderly population in China, healthcare services for the elderly have gradually become an important welfare resource. However, the healthcare service for the elderly still has problems such as mismatched supply and demand and unbalanced resources. In order to effectively eliminate the path barriers to match supply and demand, and improve the accessibility of healthcare services, this paper introduces the sustainability of the healthcare service based on the accessibility theory, and constructs an index system from the three dimensions of potential accessibility, realized accessibility, and sustainable accessibility of healthcare services for the elderly. Then, the paper makes a practice application of the index system based on survey data of healthcare services from Shaanxi province, China. Finally, the paper finds that the total accessibility and sustainable accessibility of healthcare services for the elderly in Shaanxi Province are at an average level. The score of potential accessibility is high, indicating that elderly people have greater opportunities to use healthcare services. The realized accessibility score is low, which indicates that the actual use of healthcare services for the elderly presents low satisfaction.

## 1. Introduction

By the end of 2019, the Chinese population of 60 years old and above was close to 254 million people, accounting for 18.1% of the total population of China. Among them, there are 176 million people aged 65 and over, accounting for 12.6%. With the continuous increase of the elderly population, the demand of healthcare services for the elderly is becoming more and more urgent [1]. However, at present, the number of healthcare beds for the elderly is only 29.15 per 1000 of the elderly population in China. Among them, 4.292 million beds are in healthcare institutions and 3.24 million in the community, far below international standards, and the vacancy rate of healthcare beds for the elderly is as high as 20%. There is a serious waste of resources and urban–rural differences. The reason is that there are certain path barriers to match the demand with the supply of healthcare services for the elderly [2,3]. Therefore, the improvement of healthcare services for the elderly must focus on the matching path of supply and demand and emphasize the accessibility of healthcare services for the elderly. Only by establishing a bridge between the supply and demand of healthcare services can the satisfaction and sense of acquisition of the elderly be effectively improved, and the construction of the system of healthcare services for the elderly be accelerated.

In recent years, the Chinese government has attached great importance to the accessibility of healthcare services for the elderly. In 2014, the “Notice on Promoting the Construction of Urban Healthcare Service Facilities for the Elderly” (No. 116) pointed out we should strengthen the construction of urban healthcare service facilities for the elderly, and establish and improve the urban healthcare service network to meet the needs of the elderly. In 2016, the “Notice on Supporting the Integration and Transformation of Idle Social Resources to Develop Healthcare Service for the Elderly” (No. 179) emphasized that it was necessary to fully use idle social resources and increase services to improve the accessibility of healthcare services for the elderly. In 2017, the “Notice on Carrying out Special Actions for the Construction of Nursing Home Service Quality” (No. 51) asserted that it was necessary to establish a Nursing home development mechanism oriented by quality and benefit to create affordable and good nursing homes for the elderly. In 2019, the “Opinions of the General Office of the State Council on Promoting the Development of Healthcare Service for the Elderly” (No. 5) announced that it was necessary to break through the blocking points, to remove developmental obstacles, and effectively improve the sense of acquisition, happiness, and security of the elderly and their children.

However, current research on the content of the accessibility of healthcare services for the elderly is very rare. Most scholars mainly focus on the practical utilization of healthcare services for the elderly, ignoring the sustainability of the services, which causes obviously adverse effects on promoting the healthy development of healthcare services for the elderly. As an important manifestation of people’s livelihood and well-being, the accessibility of healthcare services for the elderly is closely related to economic development, social progress, and cultural changes. Different stages of material sharing and spiritual feelings will affect the perception of accessibility for the elderly. Therefore, analyzing the accessibility of healthcare services for the elderly can fully grasp the development direction and construction goals of healthcare services for the elderly from the sustainable perspective, which has certain practical significance for breaking the barriers to the supply and demand of healthcare services for the elderly. So, how should the accessibility of healthcare services for the elderly be defined? What are the main factors? How can sustainability of the healthcare service for the elderly be measured? These issues are worthy of our discussion.

First, this article examines the concept of accessibility, emphasizes the sustainability of healthcare services for the elderly, and points out the conceptual connotation of the accessibility of healthcare services for the elderly. Then, it elaborates on the main factors and establishes an indicator system from the three dimensions of potential accessibility, realized accessibility, and sustainable accessibility. Finally, based on the survey data from Shaanxi Province, the paper calculates the indicator system of healthcare services for the elderly to measure the level of accessibility.

## 2. Literature Review

Regarding the research on the concept of “accessibility” of healthcare services, Anderson pointed out that the accessibility of public healthcare was an equal service for people regardless of economic conditions [4]. In order to further clarify the concept of accessibility, different scholars had proposed different concepts of accessibility. Roy defined accessibility as the ability to reach, obtain, or afford entrance to services [5]. Salkever believed that accessibility refers to an opportunity of people who have not yet entered the service system, and emphasized that there were differences in accessibility between people or groups [6]. Aday defined accessibility as a measure of potential or actual entry into the service system from the perspective of service use [7]. Penchansky and Thomas defined accessibility as the degree of fit between customers and the system, and divided accessibility into five dimensions: availability, reachability, acceptability, affordability, and accommodation [8]. Goddard believed that accessibility is an opportunity to reach and obtain appropriate services when services are needed. It is closely related to the situation and emphasizes the fairness of accessibility [9]. Peters emphasized the importance of quality in accessibility, and divided accessibility into quality, geographic accessibility, acceptability, and financial accessibility [10]. Levesque et al. regarded accessibility as determining medical service needs, seeking medical services, reaching medical service centers, obtaining or using medical services, and the chance of services actually provided to meet medical and health needs [11]. With the continuous improvement of the concept of accessibility, research on accessibility has gradually penetrated from healthcare to healthcare services for the elderly [12], financial services [13], and other fields.

Regarding the research on the accessibility of healthcare services for the elderly, Jadhav analyzed the accessibility of public healthcare for the elderly in rural areas and pointed out that nursing costs, human resources, and the health system are the main factors affecting accessibility [14]. Huang analyzed the relationship between the spatial accessibility of basic medical care for the elderly and the hospitalization and the emergency rates and pointed out that the spatial accessibility is an important factor affecting medical services [15]. Ford pointed out that the economic conditions of the elderly in rural areas are a key factor affecting personal and community medical services from the perspective of constructing an access path of patient medical services [16]. There are also some scholars discussing the accessibility of healthcare services for the elderly from a sustainable perspective. Mosca et al., based on European Union (EU) healthcare services for the elderly, pointed out that there were market failures of private funds and national fiscal imbalance during the long-term care services [17]. In order to provide services effectively, it is necessary to solve the sustainability of long-term care. Xu analyzed the cost of long-term care services for the elderly and pointed out that the future design of long-term care systems must consider its sustainable development capabilities [18].

Some scholars also pay attention to the research on the accessibility of the Internet, public transportation, and other aspects for the elderly. Hodge analyzed the degree of interaction between the elderly and network operators, and pointed out that the degree of matching between the elderly in rural areas and Internet services is low [19]. Shrestha analyzed the impact of public transportation on the quality of life of the elderly from the perspective of affordability, usability, acceptability, and accessibility based on the mobility of the elderly [20]. Suurmond et al. conducted interviews with ethnic minority elderly people and pointed out that language and culture were the main obstacles to the accessibility of home care [21].

Regarding the research on the index system of the accessibility, many scholars used the “5A” analysis framework proposed by Penchansky and Thomas. Shrestha described the accessibility of older people using public transport from four main issues of affordability, availability, geographic accessibility, and acceptability [20]. Van conducted an in-depth investigation into the issues of accessibility to healthcare services by older Australians from five aspects of availability, geographic accessibility accommodation, affordability, and acceptability [22]. Israel explored financial accessibility of healthcare based on four factors of availability, geographic accessibility, affordability, and acceptability [23]. Baxter conducted a brief qualitative study of accessibility to healthcare services for non-communicable diseases from the three aspects of availability, affordability, and acceptability in Mosul, Iraq [24]. In addition to academic exploration, in January 2019, the government of Chaoyang District in Beijing launched the first document called “The Evaluation Standard of Home Renovation to Fit”, which aims to evaluate the accessibility of healthcare services for the elderly in terms of daily life ability, mental state, perception and communication, and social participation.

In summary, although the research on the accessibility of healthcare services for the elderly involves many aspects, such as geography, economy, and resources, most of the studies mainly focus on the access to public healthcare for the elderly, and lack the theoretical connotation and dimensions analysis of the access to healthcare services for the elderly. In addition, for the evaluation research on the access of healthcare services for the elderly, most scholars just apply the “5A” indicators of healthcare, and lack the reflection of the uniqueness of healthcare services for the elderly and the local situation. Therefore, based on the research evolution of access, this article constructs an access analysis dimension and measurement system suitable for healthcare services for the elderly, and applies them in practice.

## 3. Analysis Framework and Index System

### 3.1. Analysis Framework

There are two main analysis dimensions of accessibility. One is the degree of fit between the user and the service system, and the other is the use and utility of the service [25]. Based on the perspective of demand, the article analyzes accessibility from the perspective of service utility.

Donabedian first involved the concept of geographic accessibility, pointing out that geographic accessibility is mainly about the location of the service and the cost of time, distance, and use of the user in the process of searching for services, and divided accessibility into geographic accessibility and social organizations accessibility [26]. Penchansky and Thomas divided accessibility into five dimensions, availability, geographical accessibility, acceptability, affordability, and accommodation [8]. Therefore, although there is no special literature that divides accessibility into geographic accessibility and social accessibility, accessibility studies are mainly divided into two dimensions, geographic accessibility and social accessibility, from the perspective of the research content of these articles.

Aday defines “accessibility” as a measure of potential or actual entry into the service system for a given population group, based on the dimension of service use [7].Accessibility is emphasized as the result of a process, determined by the interaction between the characteristics of the service system in a specific area and the characteristics of potential users. The service system adjusts its own characteristics according to the characteristics of potential users and provides related services, which means that potential accessibility of both spatial and social nature is offered to potential users. However, whether potential users can actually use or enter the system depends on obstacles that affect the characteristics of both service system and potential users. So, accessibility can be divided into potential accessibility and realized accessibility. Potential accessibility is mainly related to the availability of service resources relative to their needs for service, while realized accessibility is related to the actual use of service resources.

In order to further refine the content of accessibility, Khan and Bhardwaj introduced the analysis dimensions of opportunity and cost on the basis of Aday [27]. Accessibility opportunity refers to the process of searching for services, and accessibility cost refers to the expenditure associated with the process of searching for services. Therefore, the current accessibility analysis dimension can be expressed as a matrix, as shown in Table 1.

**Figure 1:**
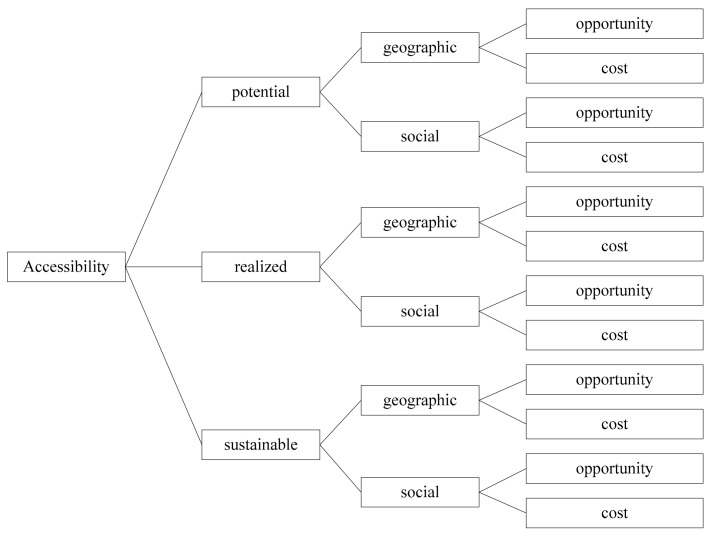
The analysis dimensions of the accessibility of healthcare services for the elderly.

Taking into account the particularity of healthcare services for the elderly, it is a long-term continuous process, whose sustainability mainly includes the continuity of healthcare service resources, the continuity of healthcare service targets, and the continuity of the healthcare service system [28]. Therefore, this article introduces sustainable accessibility on the basis of Khan and Bhardwaj and defines the accessibility of healthcare services for the elderly as the ability and utility of the elderly to use the healthcare service system. It is divided into three categories, potential accessibility, realized accessibility, and sustainable accessibility. The specific analysis dimensions are shown in Figure 1.

The potential accessibility means that the elderly have the opportunity to use healthcare services. This is mainly related to the distance between the aged users and the healthcare service supply center, the cost of discovering and searching for healthcare services, the degree of understanding of the aged users about healthcare services, and the degree of their own demand for healthcare services. The potential accessibility of healthcare services for the elderly is mainly related to the healthcare service supply system. The more adequate and balanced the supply, the greater the potential accessibility.

The realized accessibility refers to the actual use of healthcare services for the elderly. This is mainly related to the cost of the healthcare service, the waiting time for the elderly to use the healthcare service, the degree of trust the elderly have in the healthcare service center, and the degree of satisfaction in using the healthcare service. The realized accessibility of healthcare services for the elderly mainly depends on the subjective feelings of the elderly in using healthcare services. The richer the resources of healthcare services, the higher the quality and the greater the realized accessibility.

The sustainable accessibility mainly refers to the possibility of continuously using healthcare services for the elderly. This is mainly related to the health status of the elderly, the willingness to participate in social activities, the support of children and the government to the elderly, and the convenience of door-to-door care. With the gradual aging of the elderly population, the more service content and social support and the higher the sustainable accessibility.

### 3.2. Index System

Based on the analysis dimensions above, we can construct principles of the index system. The accessibility of healthcare services for the elderly is rich in connotation, and it is difficult to use a single indicator to assess it. Therefore, the establishment of an index system for the accessibility of healthcare services for the elderly should be based on the principles of scientific, concise, and transparent statistics, and at the same time, firmly grasp the humanistic connotation of the accessibility of healthcare services for the elderly, and fully reflect the heterogeneity, stability, and dynamics of the index system.

On the heterogeneity of the index system, an index system which is reasonably constructed for the accessibility to healthcare services for the elderly should allow different entities to independently choose suitable development paths. In China, there are a large number of elderly people, and they are distributed in a wide range of areas. Considering the existence of differences in ethnic characteristics, cultural traditions, and regional resources, the healthcare service for the elderly built in different regions should be heterogeneous. If a unified and a homogeneous index system is constructed for the accessibility of healthcare services for the elderly, although it is conducive to compare between different regions, it lacks rationality and fairness to a large extent. Therefore, the heterogeneity of the index system for the accessibility of healthcare services for the elderly requires considering the commonality of the evaluation objects and the regional individuality. In the index system, regional advantages should be discovered, and a distinctive healthcare service index should be explored.

On the stability of the index system, with the increasing degree of aging, the construction of healthcare services for the elderly will be a long-term task for China at present and in the future, and the accessibility index system of healthcare services for the elderly should play a stable role. The stability of the index system is not only conducive to the vertical comparison, it is also conducive to the policy formulation of healthcare services for the elderly. At the same time, it can coordinate the government, market, social, and other major forces, and reflect the sustainable construction of healthcare services for the elderly. In addition, due to the particularity of healthcare services for the elderly, the services need long-term and stable supply, such as the psychological care supply for the elderly and the supply of home care personnel. Therefore, the accessibility index system of healthcare services for the elderly needs to keep a certain degree of stability.

On the dynamics of the index system, the healthcare service for the elderly is a continuous and dynamic construction process that will change with changes in social economy and information technology. The healthcare service for the elderly has undergone the evolution of various models such as family care, social care, home and community care, mutual assistance care, smart care, and so on, and the content of healthcare services is becoming more and more diverse. Therefore, the accessibility index system of healthcare services for the elderly should be dynamic. In addition, it should be noted that dynamics and stability are not contradictory, and are the organic unity of the index system. It is necessary to maintain stability for the classification principle of the index level, and the indicators of each specific index should be dynamic and be adjusted in time with social changes.

Based on the above analysis dimensions of the accessibility of healthcare services for the elderly and the construction principles of the index system, this paper establishes an accessibility index system of healthcare services for the elderly that includes potential accessibility, realized accessibility, and sustainable accessibility, according to the existing research results of accessibility of healthcare services for the elderly and the construction practice of local government [29,30,31]. The index system includes 3 first level indicators, 12 secondary level indicators, and 23 measurement indicators, as shown in Table 2. Among them, the indicators assessing method adopts the 5-point Likert scale method and assigns values of “1, 2, 3, 4, 5” according to the accessibility from weak to strong. Elderly people perform evaluation on each indicator according to their actual feelings.

This index system, including the access opportunities, utility, and sustainability of healthcare services for the elderly, can fully reflect the development status of the accessibility of home care services, and is suitable for the quality evaluation of home-based healthcare services in China. Regarding the differences in various regions, it depends on the selection of the measurement indicators, which can be selected according to the principles of heterogeneity and dynamics, with reference to local characteristics, and economic and social changes.

## 4. Empirical Analysis

Based on the actual situation and characteristics of home-based healthcare services for the elderly in Shaanxi Province, this paper applies the accessibility index system to measure the accessibility of healthcare services for the elderly in Shaanxi Province.

### 4.1. Data Resource

The research data in the article comes from the survey conducted in July 2019. The survey team was composed of more than 20 teachers, doctoral students, and master students from Xi’an Jiaotong University. We adopted stratified sampling. The first stage of stratification is based on cities, the second stage of stratification is based on counties, the third stage of stratification is based on townships, and the fourth stage of stratification is based on villages. The fifth stage of stratification takes the elderly as a sample. Finally, we investigated three representative regions of Hanzhong, Baoji, and Yan’an, which are characterized by high aging and rapid development of healthcare services for the elderly. The authors’ survey team interviewed 300 elderly people in 2 counties (districts) in Hanzhong City. In the end, the team obtained a total of 948 valid questionnaires, which was representative of the population. The results of the survey are shown in Table 3.

Since the sample includes various elderly people of different backgrounds and different education experience, some questions involved in the questionnaire are missing answers. Taking into account the accuracy of data analysis, 348 obviously missing data were excluded. Other data were all included in the analysis model. Some missing answers were estimated through iteration based on known information, to ensure statistical integrity to the greatest extent. Finally, the basic descriptive statistics of each index are shown in Table 4.

It can be seen from Table 4 that the basic statistical indicators of most indexes are within the normal range. Among them, the standard deviation is close to 1, indicating that the data is concentrated near a certain central value, and the kurtosis and skewness are mostly between −1 to 1, indicating that the data is close to normal distribution. It shows that the questionnaire has no outliers and has certain reliability.

### 4.2. Results

This article used SPSS20.0 as an analysis tool to calculate weights for different indicators based on principal component analysis. First, the reliability of the data was tested, and the validity of the questionnaire is 99.2%, indicating that its inherent consistency is high. The overall Alpha coefficient of the questionnaire is 0.863, indicating that the questionnaire data is reliable. Then, the Kaiser-Meyer-Olkin (KMO) and Bartlett tests were performed, as shown in Table 5. The KMO value is 0.858, which is greater than 0.5, indicating that the correlation between the variables is high and the factor analysis is feasible. Bartlett’s test is significant at the level of 0.000, the hypothesis of the independence of the variables is not established, and the applicability test of factor analysis is passed.

The common factors were extracted by principal component analysis. As shown in Table 6, 6 factor variables with eigenvalues greater than 1 can be obtained, and the cumulative variance contribution is 63.110%, indicating that the first 6 common factors extracted have a greater impact on the accessibility of healthcare services for the elderly.

The rotation component matrix, obtained after the variance maximization orthogonal rotation of the factor loading matrix, is shown in Table 7, which can better reflect the relationship between the variables of the common factors.

According to the proportion of variance contribution rate of each common factor to total variance contribution rate and the eigenvalue after rotation, the objective weight of each indicator is determined in Table 8.

From the perspective of the weights of various indicators, the total weight of realized accessibility is 0.3740. Among the top ten indicators, there are six realized accessibility indicators, indicating that realized accessibility accounts for a large proportion of the accessibility of healthcare services for the elderly, which shows that the elderly pay more attention to the use process of healthcare services, especially the frequency and convenience of healthcare services. Realized accessibility requires healthcare service providers to pay attention to the geographical accessibility of healthcare services, and to emphasize quality accessibility and utility accessibility of healthcare services for the elderly. The total weight of potential accessibility is 0.2960. Among them, the two indicators of understanding and attention to healthcare services have a larger proportion, indicating that the potential accessibility of healthcare services is mainly related to the availability of information. Therefore, the government and the community must popularize the knowledge of healthcare services, increase publicity for healthcare services, and timely update healthcare service information for the elderly. The total weight of sustainable accessibility is 0.3300. Among them, the capacity and degree of care of the door-to-door healthcare service are relatively large, indicating that the sustainability of healthcare services is undergoing a transformation from demand-led to supply-led. Therefore, increasing the individualization and diversification of the supply of healthcare services will help improve the sustainable accessibility of healthcare services for the elderly.

### 4.3. Evaluation

Taking into account the complexity of the index system and the uncertainty of subjective evaluation, the use of fuzzy comprehensive evaluation can effectively evaluate the comprehensive level of accessibility of healthcare services for the elderly.

Among them, the evaluation level of the accessibility of healthcare services is $V=V_{i}\left(i=1,2,\;\ldots 5\right)$, indicating the degree of accessibility. The index level contains 23 measurement indicators, so $U=U_{j}\left(j=1,2,\;\ldots 23\right)$, the degree of membership, $R_{j}$, is the ratio of the number of persons belonging to the evaluation level, $V_{i}$, of each indicator, $U_{j}$, to the total number of surveys. Therefore, the score of each indicator can be obtained in Table 9.

Finally, the final score of the accessibility of healthcare services for the elderly can be obtained by the sum of multiplying the weight of each indicator in the total accessibility and the indicator score. Similarly, the final scores of potential, realized, and sustainable accessibility of healthcare services can be obtained respectively by the sum of multiplying the weight of each indicator in the sub-accessibility and the indicator score.

The total accessibility score of healthcare services is 3.24, indicating that the total accessibility of healthcare services for the elderly in Shaanxi is average, which indicates that the ability and the utility to use healthcare services need to be improved. From the perspective of demand of healthcare services for the elderly, the accessibility of healthcare services is related to the elderly’s ability to use healthcare services. The elderly’s ability to perceive, recognize, obtain, and pay for healthcare services is still relatively poor, which is closely related with the living habits, education experience, economic status, and social sensitivity of the elderly. From the perspective of supply of healthcare services for the elderly, the accessibility of healthcare services is related to the mode, content, quality, and frequency of supply of healthcare services, which indicates that the use of healthcare resources has not yet effectively matched the needs of the elderly, but has only extensively increased the resources of healthcare services, ignoring the pertinence and efficiency of the healthcare services for the elderly resources.

The potential accessibility score is 3.52, which is higher than the total accessibility score of healthcare services, indicating that the elderly have greater opportunities to use healthcare services and there is a certain demand market for healthcare services. The high score for the potential accessibility of healthcare services is related to the policy proposed by Shaanxi Province to create a 15min healthcare service circle in 2019, because the potential accessibility is mainly reflected in the convenience of information, policy support, and geographic accessibility of healthcare services for the elderly. In terms of potential accessibility, the score of completeness of the healthcare service information platform is low, indicating that the potential accessibility still has problems such as untimely information sharing and inadequate publicity of relevant policies. With the continuous development of Internet technology and the continuous improvement of information platforms, the potential accessibility can be greatly improved, and the consumer market of healthcare services for the elderly will be greatly stimulated.

The realized accessibility score is 3.01. The realized accessibility index has the largest weight, but the score is the lowest, indicating that the actual use of healthcare services is not ideal. From the perspective of healthcare service content, the scores for the content and the resource adequacy of healthcare services are low, indicating that the single service content and the shortage of resources have greatly affected the satisfaction of the elderly. From the perspective of the usage of healthcare services, the higher willingness to use is consistent with the higher potential accessibility score, indicating that the elderly have a higher demand for healthcare services. From the perspective of healthcare service forms, self-seeking services in community centers is the main form for the elderly, which greatly reduce the accessibility of healthcare services for the elderly. With the full coverage of the Internet and information technology, healthcare services for the elderly must also implement various forms of “Internet +” services to improve the convenience.

The sustainable accessibility score is 3.26, which is consistent with the total accessibility score, indicating that the sustainability is related to the comprehensive level of healthcare services. From the scores of various indicators of sustainable accessibility, the scores of door-to-door healthcare are low, which indicates that the service suppliers lack active understanding of the service needs of the elderly, which is not only detrimental to the sustainable accessibility of healthcare services, but also fails to achieve universal accessibility of disability, dementia, and other special elderly needs. The scores of the social participation for the elderly and the prospects of healthcare services are high, indicating that the elderly are willing to use healthcare services in the community and the potential of sustainable healthcare services for the elderly is great. In short, sustainable accessibility is a good indicator for the comprehensive, coordinated, and dynamic development of healthcare services.

Therefore, based on the above analysis, the improvement of the accessibility of healthcare services for the elderly must not only solve the practical development dilemma of realized accessibility, but also break down the service barriers of potential accessibility and coordinate the overall sustainable accessibility. In response to the above problems, the following suggestions are made. First, the construction of the healthcare service information platform needs to be improved to cover all elderly people as much as possible. Healthcare service resources should be coordinated all over the country to increase the potential accessibility of healthcare services for the elderly. Second, the government should increase healthcare service content, make full use of the Internet, broaden healthcare service channels, effectively allocate healthcare service resources by market, and determine resource allocation based on the actual needs of the elderly, to improve the realized accessibility of healthcare services for the elderly. Third, the healthcare centers should increase the form of door-to-door services for the elderly, actively focus on the actual needs of elderly, ensure the basic welfare of the elderly, and stimulate the consumer market for the elderly, to realize the sustainable accessibility of healthcare services for the elderly.

## 5. Conclusions

Based on the demand of healthcare services for the elderly and the theoretical evolution of accessibility, this article explained the concept of accessibility from the perspectives of potential accessibility, realized accessibility, and sustainable accessibility. So, this article broadens the theory of accessibility from the perspective of sustainability and proposed an evaluation system for home-based healthcare services. The main conclusions are as follows.

First, the accessibility of healthcare services for the elderly includes not only access opportunity and realized use, but also the sustainability of healthcare services, which meets the needs of healthy aging.

Second, this article proposed an evaluation index system for healthcare services from three aspects of potential accessibility, realized accessibility, and sustainable accessibility, which can comprehensively and systematically assess the level of healthcare services, and is suitable for the quality evaluation of home-based healthcare services for the elderly in China.

Third, the total accessibility of healthcare services in Shaanxi Province is average, the potential accessibility score is high, the realized accessibility score is low, and the sustainable accessibility is consistent with the total accessibility score. This is mainly due to the fact that Shaanxi Province has not yet formed a mature healthcare service market, the consumption consciousness of the elderly has not yet formed, and healthcare services are still in a stage of oversupply.

## Figures and Tables

**Table 1:** The Khan model of accessibility.

Accessibility	Geographic		Social	
Potential	Opportunity		Opportunity	
		Cost		Cost
Realized	Opportunity		Opportunity	
		Cost		Cost

**Table 2:** Accessibility index system of healthcare services for the elderly.

Total Index	First Level	Secondary Level	Measurement Indicators (Number)
Accessibility index system of healthcare services for the elderly	Potential accessibility	Geographic accessibility opportunity	Satisfaction with community life (1)
			Convenience to community healthcare service center (2)
		Geographic accessibility cost	Reasonable level of expenditure for basic healthcare service (3)
		Social accessibility opportunity	Understanding of healthcare service (4)
			attention to healthcare service (5)
		Social accessibility cost	Perfection of the information platform for healthcare service (6)
			Frequency of policy publicity for healthcare service (7)
	Realized accessibility	Geographic accessibility opportunity	Completeness of the content of healthcare service in community (8)
			Adequacy of resources of healthcare service in community (9)
		Geographic accessibility cost	Willingness to use healthcare service (10)
			Frequency of use of healthcare service (11)
		Social accessibility opportunity	Trust in using healthcare service (12)
			Benefits of using healthcare services for the elderly (13)
		Social accessibility cost	Convenience of using healthcare services for the elderly (14)
			Waiting time for healthcare service (15)
	Sustainable accessibility	Geographic accessibility opportunity	Ability to solve problems of healthcare personnel (16)
			Trust of healthcare personnel in door-to-door care for the elderly (17)
		Geographic accessibility cost	Timeliness of door-to-door care (18)
			Caring for the elderly by healthcare personnel (19)
		Social accessibility opportunity	Willingness of elderly people to participate in social activity (20)
			Health status of elderly people (21)
		Social accessibility cost	Prospects of healthcare services for the elderly in community (22)
			Economic pressure on healthcare services for the elderly (23)

**Table 3:** Descriptive statistics of the sample.

Individual Characteristics	Frequency	Percentage (%)
Gender		
Male	405	42.7
Female	543	57.3
Age		
60–69	415	43.8
70–79	325	34.3
Above 80	208	21.9
Registered permanent residence		
City	518	54.6
Countryside	430	45.4
Political status		
Communist Party	247	26.1
The masses	689	72.7
Democratic parties	12	1.3
Health status		
Very bad	43	4.5
Bad	179	18.9
Average	249	26.3
Better	330	34.8
Well	147	15.5
Education		
Elementary	455	48.0
Junior	249	26.3
High	171	18.0
Junior college	49	5.2
Bachelor	24	2.5

**Table 4:** Basic descriptive statistics of each index.

Indicator	Mean	Median	Standard Deviation	Kurtosis	Skewness
1	4.52	5	0.69	1.00	−1.28
2	4.59	5	0.87	4.56	−2.25
3	3.75	4	1.46	−0.56	−0.94
4	3.64	4	1.31	−1.00	−0.55
5	3.24	3	1.31	−1.15	−0.18
6	2.15	2	1.18	−0.12	0.89
7	3.20	3	1.30	−1.02	−0.23
8	2.74	3	1.22	−0.90	0.29
9	3.04	3	1.13	−0.85	−0.03
10	2.95	3	1.19	−0.76	0.05
11	3.62	4	1.17	−0.56	−0.62
12	3.64	4	1.30	−0.51	−0.76
13	2.99	3	1.39	−1.24	−0.12
14	3.30	3	1.04	0.11	−0.54
15	2.08	1	1.50	−0.71	0.96
16	2.86	3	1.14	−0.61	0.10
17	3.58	4	1.30	−0.63	−0.68
18	3.05	3	1.19	−0.89	−0.24
19	2.60	2	1.27	−0.92	0.36
20	3.47	4	1.29	−0.81	−0.54
21	3.45	4	1.10	−0.78	−0.25
22	3.11	3	1.40	−1.35	−0.14
23	4.07	4	1.13	0.56	−1.13

**Table 5:** The Kaiser-Meyer-Olkin (KMO) and Bartlett’s tests.

**Kaiser-Meyer-Olkin Measure with Adequacy Sample**		0.858
Bartlett’s sphericity test	Approximate chi-square	5836.174
	Degrees of freedom.	253
	Significance	0.000

**Table 6:** Total variance explained.

Common Factors	Initial Eigenvalues			Rotating Sum of Squares Loading		
	Total	Variance (%)	Cumulated (%)	Total	Variance (%)	Cumulated (%)
1	6.379	27.734	27.734	3.685	16.020	16.020
2	2.870	12.479	40.213	3.404	14.801	30.821
3	1.559	6.778	46.991	2.261	9.831	40.652
4	1.298	5.643	52.634	1.950	8.480	49.132
5	1.247	5.424	58.058	1.832	7.966	57.097
6	1.162	5.052	63.110	1.383	6.013	63.110

**Table 7:** Rotation component matrix.

Index	Component 1	Component 2	Component 3	Component 4	Component 5	Component 6
1	0.011	0.078	0.197	0.017	0.070	0.406
2	−0.004	0.107	0.032	0.796	0.203	−0.043
3	0.158	−0.183	0.509	−0.028	−0.344	0.155
4	0.128	0.174	0.169	0.207	0.742	0.099
5	0.099	0.172	0.165	0.160	0.808	0.057
6	0.114	0.766	−0.037	0.202	0.189	0.036
7	0.175	0.553	0.057	0.113	0.207	0.250
8	0.157	0.747	0.133	0.309	0.006	0.052
9	0.019	0.683	0.260	0.302	−0.079	0.097
10	0.167	0.459	0.560	0.259	0.200	0.043
11	0.111	0.226	0.738	0.068	0.138	0.043
12	0.912	0.003	0.033	0.055	0.022	0.065
13	−0.053	0.380	0.081	0.766	0.160	0.007
14	0.289	0.493	0.375	−0.028	0.111	−0.160
15	0.110	0.631	0.114	−0.244	0.327	−0.121
16	0.793	0.295	0.135	−0.088	0.078	0.041
17	0.901	−0.034	0.093	0.015	0.022	0.031
18	0.859	0.196	0.151	−0.040	0.032	0.051
19	0.626	0.407	0.021	0.124	0.195	0.145
20	0.028	0.093	0.690	0.008	0.255	0.062
21	0.077	0.067	−0.030	−0.095	0.194	0.718
22	0.084	−0.036	−0.009	0.056	−0.168	0.712
23	0.093	0.145	0.520	0.498	−0.005	0.039

**Table 8:** Weight of indicator of accessibility of healthcare services for the elderly.

Sub-Accessibility	Measurement Indicators	Percentage of Total Accessibility Weight (Rank)	Percentage of Sub-Accessibility Weight
Potential Accessibility	1	0.0256 (23)	0.0864
	2	0.0389 (17)	0.1314
	3	0.0452 (11)	0.1527
	4	0.0499 (3)	0.1684
	5	0.0479 (5)	0.1620
	6	0.0441 (13)	0.1490
	7	0.0445 (12)	0.1502
Realized Accessibility	8	0.0461 (10)	0.1232
	9	0.0473 (8)	0.1264
	10	0.0554 (1)	0.1481
	11	0.0435 (15)	0.1162
	12	0.0358 (21)	0.0956
	13	0.0475 (7)	0.1270
	14	0.0478 (6)	0.1278
	15	0.0508 (2)	0.1358
Sustainable Accessibility	16	0.0469 (9)	0.1422
	17	0.0360 (20)	0.1090
	18	0.0436 (14)	0.1322
	19	0.0498 (4)	0.1510
	20	0.0373 (19)	0.1130
	21	0.0388 (18)	0.1175
	22	0.0350 (22)	0.1059
	23	0.0427 (16)	0.1293

**Table 9:** Score of indicators of the accessibility of healthcare services for the elderly.

Potential Accessibility Indicators	Score	Realized Accessibility Indicators	Score	Sustainable Accessibility Indicators	Score
1	4.52	8	2.74	16	2.86
2	3.75	9	3.04	17	3.58
3	4.59	10	2.95	18	3.05
4	3.64	11	3.62	19	2.60
5	3.24	12	3.64	20	3.47
6	2.15	13	2.99	21	3.45
7	3.20	14	3.30	22	3.11
		15	2.08	23	4.07
